# Genome-wide association study of emotional empathy in children

**DOI:** 10.1038/s41598-020-62693-6

**Published:** 2020-05-04

**Authors:** M. R. Woodbury-Smith, A. D. Paterson, P. Szatmari, S. W. Scherer

**Affiliations:** 1 Translational and Clinical Research Institute, Newcastle University, Newcastle upon Tyne, UK; 2 The Centre for Applied Genomics, The Hospital for Sick Children, Toronto, ON Canada; 3 Division of Epidemiology and Biostatistics, Dalla Lana School of Public Health, University of Toronto, Toronto, ON Canada; 4 Centre for Addiction and Mental Health, The Hospital for Sick Children & University of Toronto, Toronto, ON Canada; 5 McLaughlin Centre and Department of Molecular Genetics, University of Toronto, Toronto, ON Canada

**Keywords:** Quantitative trait, Prefrontal cortex

## Abstract

The genetic contribution to different aspects of empathy is now established, although the exact loci are unknown. We undertook a genome-wide association study of emotional empathy (EE) as measured by emotion recognition skills in 4,780 8-year old children from the ALSPAC cohort who were genotyped and imputed to Phase 1 version 3 of the 1000 Genomes Project. We failed to find any genome-wide significant signal in either our unstratified analysis or analysis stratified according to sex. A gene-based association analysis similarly failed to find any significant loci. In contrast, our transcriptome-wide association study (TWAS) with a whole blood reference panel identified two significant loci in the unstratified analysis, residualised for the effects of age, sex and IQ. One signal was for *CD93* on chromosome 20; this gene is not strongly expressed in the brain, however. The other signal was for *AL118508*, a non-protein coding pseudogene, which completely lies within CD93’s genomic coordinates, thereby explaining its signal. Neither are obvious candidates for involvement in the brain processes that underlie emotion recognition and its developmental pathways.

## Introduction

Our capacity to negotiate the complexity of the social world represents the remarkable evolutionary development of a wide range of mechanisms for the processing of social information. Some of our most advanced skills, such as language and empathy, mediate our interaction with our social environment^1,2^. These skills are crucial in navigating our social milieu, in terms of independent living, vocational success and wider interpersonal relationships. Empathy itself, referring to the ability to share an emotional experience with another person, has been extensively studied from a biological perspective^3^. Two dimensions of empathy are widely recognised, namely emotional and cognitive empathy^3^. The former essentially describes the process of emotional isomorphism with another person, i.e. the sharing of their emotional state. Consequently, it requires the decoding of that information from cues offered by others’ facial expressions, vocalisations, and gestures^4^. In contrast, cognitive empathy refers to the ability to understand more complex mental states, and is therefore more closely aligned with the concept of theory of mind (ToM) or mentalizing^5–7^. From an evolutionary perspective, although attachment and parental care are observed across species, empathy is only truly developed in mammals that possess self-awareness, demonstrable among primates but very few other animals. Phylogenetically, emotional empathy is older and less complex, with ToM also requiring the ability to perspective take^8^.

Using a variety of experimental paradigms research is now beginning to unravel the brain’s mechanisms for empathy. For example, much progress has been made in delineating the underlying neuropsychological dimensions of both emotional and cognitive empathy using a variety of experimental paradigms^9^. These same dimensions have also been mapped onto brain networks using functional neuroimaging^10^. Although a full taxonomy is far from clear, the amygdala and its prefrontal and superior temporal connections are known to play a crucial role in the processing of social information, thereby mediating elements of empathy^10^.

Twin and family studies have demonstrated that the different aspects of empathy are heritable, but increasingly influenced by shared environmental factors as a child grows. Specifically, Hughes and Cutting^11^ calculated a heritability of 67% (95% CI: 26–79%, best fitting model χ^2^(4) = 0.79, n.s., AIC = −7.21) for cognitive empathy for 3-year old twin pairs using a false belief paradigm, but by 5 years shared environment became increasingly important^12^. Autism Spectrum Disorder (ASD), a developmental disorder in which an abnormality of social interaction is a core impairment, is also known to be principally genetic in aetiology, with more than 100 genes or genomic regions harbouring rare genetic variants implicated so far^13^. Additionally, there is emerging evidence for the role of more common variants, sometimes coupled with rare variants, consistent with the genetic landscape of other neurodevelopmental and complex medical disorders^14,15^.

Recently, efforts have also been underway to identify common genetic variants that may be associated with different aspects of social cognition. For example, Warrier and colleagues conducted a genome-wide association (GWA) study of cognitive empathy using customers from 23 and Me and subjects from the Brisbane Longitudinal Twin Study^16^. Heritability for cognitive empathy was estimated at 5.8% (95% CI: 4.5–7.2%; p = 1 × 10^−17^), with a female specific locus at 3p26.1 reaching genome-wide significance. The leading SNP and the other 21 SNPs in high linkage disequilibrium with it are near *LRRN1*, which is highly expressed in brain tissue^17^. In a separate study, Warrier and Baron-Cohen^18^ also investigated the genetics of cognitive empathy using data from the Avon Longitudinal Study of Parents and Children, a prospective birth cohort study (ALSPAC, see below). Among teenagers, no GWA significant signal was identified, and SNP-based heritability was negligible (0.13%, p = 0.16). One limitation may have been the task used to measure cognitive empathy, the ‘Triangles Task’, which, although validated for ToM^19^, has little ecological validity.

In contrast, Coleman and colleagues^20^ investigated common variants associated with emotion recognition as measured by the Diagnostic Analysis of Nonverbal Accuracy Scale (DANVA)^21^ in the same ALSPAC dataset. Theirs did not find any associated variants, and nor did they identify a heritable component from SNP based heritability. However, a potential confound in Coleman *et al*.’s analyses is population stratification, which was not fully taken into consideration. Moreover, other layers of analysis, such as gene based association and transcriptome-wide association, provide additional opportunities for identifying signals that may be otherwise not seen in GWAS.

Therefore, in this current study ALSPAC data were used to investigate emotional empathy (EE) using the Diagnostic Analysis of Nonverbal Accuracy Scale (DANVA)^21^. Specifically, we examined the common genetic architecture of EE among 8-year old males and females evaluated using an established EE paradigm and genotyped and imputed to Phase 1 version 3 of the 1000 Genomes Project^22^. Further downstream analyses, including gene based and transcriptome-wide association along with polygenic risk score predictive analysis, were also undertaken.

## Results

The total sample comprised 4,780 children (males: 2,382, females: 2,393; ratio ~ 1:1) with a mean age at time of assessment of 103.6 months (SD: 3.7 months). There was no difference between the mean ages of males and females (males: mean (SD) 104 months (3.8); females: mean (SD) 104 months (3.6). The mean (SD) IQ (full scale IQ) assessed at the same age was 105.1 (16.4) for the complete sample, with no sex difference (males: mean (SD) 105 (16.9); females: mean (SD) 105 (15.7).

The total raw DANVA score (max. 24) did not follow a normal distribution (Supplementary Fig. 1: DANVA^3^ distribution). A cubed transformation of the raw scores improved distributional properties towards normality. DANVA^3^ was therefore used as the trait measure in all subsequent analyses. Both age and IQ were significantly correlated with DANVA^3^ scores, and DANVA^3^ scores differed significantly between males and females (DANVA^3^ scores: female > male, effect size = 0.2, p = 1.4 × 10^−8^). Our principal GWAS analyses therefore examined DANVA^3^ residualized for the effects of age, sex and IQ. As a corollary, and in order to be able to directly compared results to those of Warrier *et al*.^16,18^, we also conducted analyses residualizing for only age and sex.

### Genome-wide association

**Figure 1:**
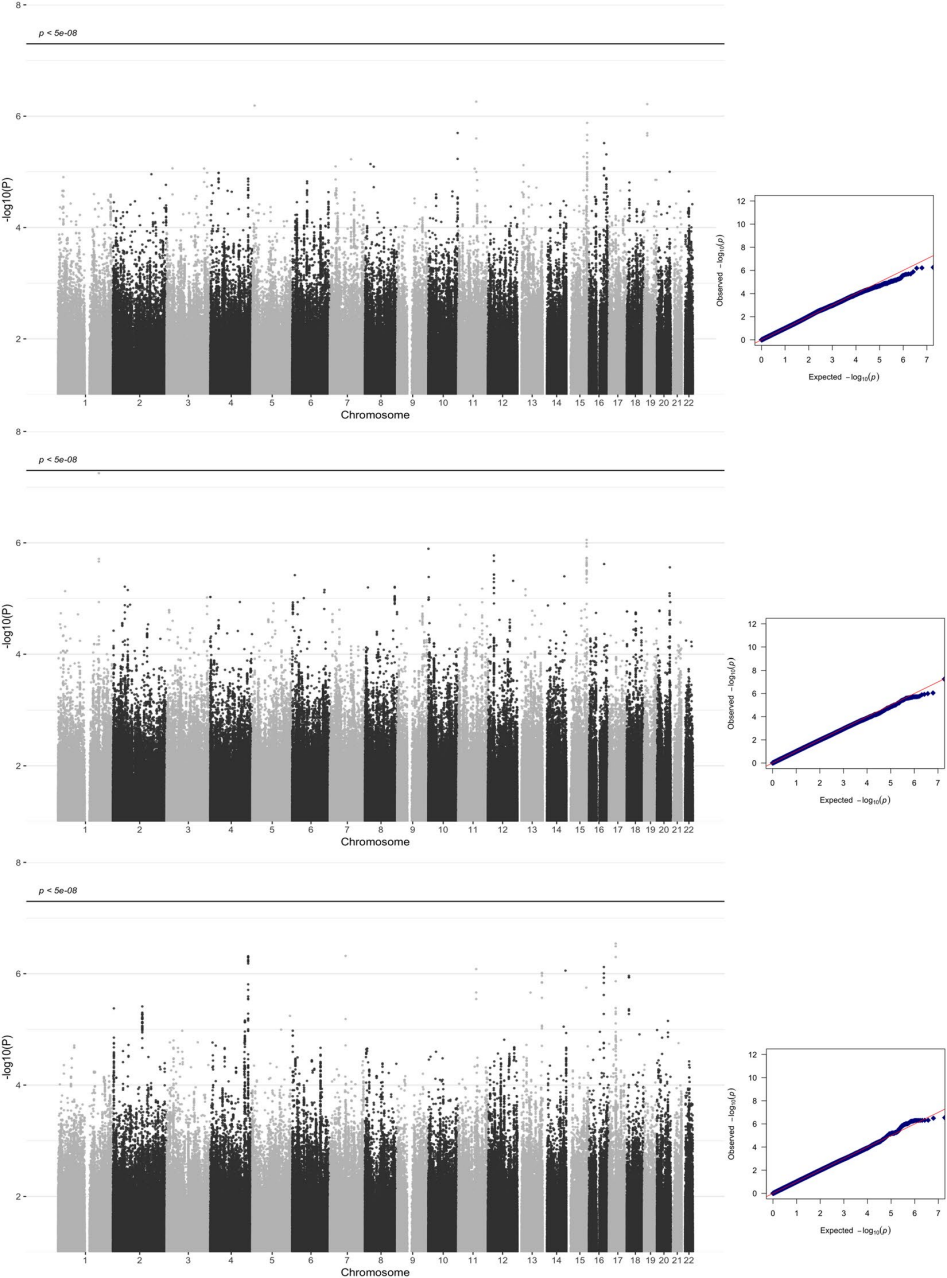
Manhattan (left) and QQ (right) plots for GWAS, unstratified (top), female only (middle), male only (bottom). Note y-axis starts at −log10(P) = 1.

All individuals were of European ancestry according to our population stratification analysis (see methods). We further investigated for confounding using LDSC^23^ using LD scores calculated from 1000 Genomes European samples. An LDSC intercept of 0.99 (SE = 0.006) was generated (lambda-GC = 0.99). This intercept of less than 1 is consistent with the absence of population stratification. A SNP heritability of 3.25% (SE 8.8%) was obtained. The results of the GWA analysis for emotional empathy across all autosomes, as measured by total DANVA^3^ score residualized for the effect of sex, age and IQ, are depicted in Fig. 1. The three analyses include total sample, and further analyses stratified by sex. These analyses did not identify any SNPs significant at the p < 5 × 10^−8^ level. In the female-only analysis the most significant locus identified was on chromosome 1 (rs12407722, β = −1151.37, se = 212.7, p = 5.6 × 10^−8^, male: β = 102.3, se = 224.5, p = 4.476e-06). This SNP lies in an intergenic region. The next most significant loci were in the male only analysis on chromosome 17 (rs2032753, β = 411.0, se = 80.2, p = 3.21 × 10^−7^, a *MYO1D* intronic variant), chromosome 4 (rs7686071, −β = 564.53, se = 111.9, p = 4.8 × 10^−7^, intergenic) and chromosome 7 (rs113791338, β = −1134.17, se = 224.2, p = 4.79 × 10^−7^, a *CALN1* intronic variant). None of these loci overlap with the Warrier *et al*. cognitive empathy loci^16^. Additionally, none overlapped the top SNPs from the Coleman *et al*. study. All SNPs with p < 1 × 10^−6^ are provided in Supplementary Table 1.

We also undertook a GWA analysis of total DANVA^3^ score residualized for the effect of sex and age. The results are shown in Supplementary Fig. 2. The GWA analysis did not identify any significant SNPs either for the complete sample or when stratified by sex. Again, none of the loci where non-significant signals were apparent overlapped the loci highlighted by Warrier *et al*.^16^ Additionally, none overlapped the top SNPs from the Coleman *et al*. study. All pruned SNPs with p < 1 × 10^−6^ are provided in Supplementary Table 2.

We additionally investigated direction of effects for Warrier *et al*.’s top 10,000 SNPs to examine concordance with our own analyses. We first LD pruned these SNPs using LDlink’s SNPclip option based on the 1000 genomes phase 3 (version 5) dataset^24^. We then examined the distribution of the p values for these same SNPs from our ALSPAC GWAS, and tested those p values for deviation from a uniform distribution using the Kolmogorov-Smirnov test. Our data were consistent with uniformity, indicating lack of directional concordance (D = 0.99, p-value = 0.38).

### Gene-based association

We next undertook a gene-based association test for a total of 15,056 autosomal genes using MAGMA [v.1]^25^. No gene passed the genome-wide threshold for significance (Bonferroni 0.05/15056 = 3.3 × 10^−6^) (Supplementary Fig. 3 [residualized for IQ, age and sex]; Supplementary Fig. 4 [residualized for age and sex]). Of the four genes with smallest p-values, three were from the male only analysis on chromosomes 6 (*ALDH5A1*, p = 2.1 × 10^−5^), 11 (*C11orf44*, p = 3.4 × 10^−5^) and 17 (*MYO1D*, 3.4 × 10^−5^). This gene was also identified by the intronic variant signal at rs2032753 in the GWAS. The other gene was from the corollary female only analysis (i.e. residualizing for only age and sex) on chromosome 12 (*TM7SF3*, p = 3.9 × 10^−5^).

### Transcriptome-wide association

We undertook a TWAS with 2057 genes using weights derived from GTEx whole blood samples [V.6] as described in Gusev *et al*.^26^. Two signals from the non-stratified analysis residualized for age, sex and IQ were significant at the Bonferroni corrected level of significance (0.05/2057 = 2.4 × 10^−4^), with under expression of the genes positively associated with emotional empathy (*CD93*, p = 7.6 × 10^−5^ and *AL118508*, p = 1.6 × 10^−4^). (Supplementary Fig. 5 [residualized for IQ, age and sex]; Supplementary Fig. 6 [residualized for age and sex]). The coordinates of these two genes indicate that *AL118508* is in fact a non-expressed pseudogene that lies entirely within the larger *CD93*. Its signal is therefore simply an artifact of the overlap with *CD93*.

### Polygenic risk

Polygenic risk scores were calculated for ASD^27^, schizophrenia^28^, bipolar disorder^29^, educational attainment^30^, cognitive performance^30^ and obsessive-compulsive disorder^31^. No correlation was observed between polygenic scores and EE for ASD, schizophrenia, bipolar disorder and obsessive-compulsive disorder across the range of P-value thresholds for the complete sample and also when covarying for sex (Supplementary Fig. 7). For emotional attainment there was evidence of significant positive correlation across a range of p-value thresholds, although only limited variance was predicted by the model (Supplementary Fig. 7). For cognitive performance, a marginally significant positive result was obtained at one p-value threshold.

## Discussion

We undertook a genome-wide association study of emotional empathy as measured by emotion recognition skills in 4,780 8-year old children. We failed to find any genome-wide significant signal in either our unstratified analysis or analysis stratified according to sex. A gene-based association analysis similarly failed to find any significant loci. In contrast, our TWAS identified two significant loci in the unstratified analysis, residualised for the effects of age, sex and IQ. *CD93* on chromosome 20 is not strongly expressed in either the adult or the developing brain (https://www.ebi.ac.uk/gxa/genes/). *AL118508*, also on chromosome 20, is a non-protein coding pseudogene, whose coordinates lie within those of AL118508, which explains its signal. Neither are obvious candidates for involvement in the brain processes that underlie emotion recognition and its developmental pathways. Given the recent suggestive findings of new potential mechanisms involved in ASD, however, these genomic regions should be further scrutinized^32–35^.

Among the non-significant findings, signals are either intergenic or overlapping genes that are not strongly brain expressed or implicated in brain development. The one exception is *MYO1D*, identified in both GWAS and gene-based association for the male only analysis. This gene encodes a widely expressed protein which is also strongly expressed in the developing brain^36^ and is in a region that has previously shown evidence of linkage to the neurodevelopmental disorder autism spectrum disorder^37^. This is particularly significant as ASD is known to be associated with impairments in the ability to decode emotional expressions in faces^10^.

None of the signals identified in our study overlap those of Warrier *et al*. in their GWAS study of cognitive empathy on the same sample, nor with the Coleman *et al*. study in the same sample. This may be unsurprising since these studies are likely underpowered based upon their sample sizes. Indeed, the importance of sample size, and the approximately linear relationship between number of identified associated loci and the size of the sample, has been previously demonstrated^38^. GWAS studies of complex disease traits have consistently demonstrated the small effect size at individual associated variants. For behavioural traits more generally, the effect sizes are smaller, and the impact of other factors such as everyday experiences (e.g. degree of socialisation) may be large. As such, larger sample sizes will be needed to fully realise the allelic spectra of these traits^38^.

The largely negative findings from our gene-based and TWAS analyses may similarly be explained by sample size, but other factors intrinsic to the methods themselves may also be relevant. For example, although we restricted our gene-based association analysis to SNPs within each gene, the method does allow extension of the gene’s coordinates upstream and downstream to incorporate surrounding SNPs if there is a good rationale for doing so. Similarly, the analysis can be restricted to only certain SNPs within each gene if, for example, this is based on known functionality of certain SNPs. The method itself uses pruned principal components such that some variance is lost, albeit only a very small amount (0.1%).

A lack of overlap between the genetics of different aspects of social cognition such as emotional and cognitive empathy may also be expected in light of the functional neuroimaging evidence of regional modularity. In particular, these imaging studies have identified different structures mediating simple and complex emotion recognition. Although there is great interest in using non-human primates for studying brain disorders, particularly in view of their highly developed social behaviour, there are currently no models that have examined genetic association or gene disruption in social behaviour^39^.

Moreover, in addition to those brain processes involved in different aspects of social cognition, the methodology of different study designs will require the recruitment of additional processes in decoding the information presented. For example, one of Warrier *et al*.’s studies of cognitive empathy used images of eyes which form part of a standardised measure of cognitive empathy. Extracting the complex emotion depicted in only the eyes might be different from the same information from the whole face. Similarly, static images may involve different cognitive processes than animated images, and social information portrayed in abstract images or cartoons may involve yet other processes. From a methodological point of view, unravelling these elements into more fundamental, orthogonal dimensions of social cognition is a challenge. Similarly, to ensure ecological validity tasks must also necessarily involve real life scenarios which tends to undermine task simplification. This dilemma in the biological research of social cognition will require more sophisticated methods of acquiring data, such as eye tracking which does overcome some of the aforementioned limitations. Similarly, brain imaging phenotypes may offer the opportunity for proxy measures of cognitive phenotypes at the psychological level^40^.

One further element confounding research into the genetics of social cognition is the developmental nature of the traits being examined. Different cognitive skills are acquired at different stages of childhood, and there is much inter-individual variation in these milestones. The longitudinal measurement of traits, using identical assessments at different points in childhood, will overcome this but does add an additional layer of complexity to study design. However, this still does not explain why our results may fail to show any overlap with Coleman *et al*.’s, which essentially is based on the same trait in the same sample. There are many relatively arbitrary decisions that need to be made when undertaking GWAS. On consideration of such factors, we note some striking differences between the two studies (Supplementary Table 3). This includes consideration of population stratification and the imputation method used, both of which may be of major importance in explaining observed differences in results.

In summary, therefore, although we failed to find any genome-wide significant signals from our GWAS, and the TWAS signals that are significant are not easily interpretable, this does not rule out an important role for genetics in the development of the skills required to decode emotions from facial stimuli. Future larger studies will need to take into consideration the methodological issues outlined above to ensure both adequate power and trait validity.

## Methods

### Participants

The Avon Longitudinal Study of Parents and Children (ALSPAC) is a longitudinal birth cohort with a sampling frame of all pregnant women living in the Avon region of the UK with expected delivery date between 1^st^ April 1991 and 31^st^ December 1992^41,42^. Initially 14,541 pregnant women were recruited, with 14,062 live births and 13,988 children who were alive at 1 year of age. Additional enrollment took place when the oldest children were 7 years of age. Questionnaires and face to face assessments have been carried out on participating children at specified time points measuring a variety of experiences, traits and developmental milestones. One such assessment discussed subsequently is the Diagnostic Analysis of Nonverbal Accuracy Scale (DANVA)^21^, which includes subtests to measure the ability to decode facial expressions. All participating children were invited to complete this at age 8 years (N = 7,488 invited). Please note that the study website contains details of all data that is available through a fully searchable data dictionary and variable search tool (http://www.bristol.ac.uk/alspac/researchers/our-data/). Ethical approval for the study was obtained from the ALSPAC Ethics and Law Committee and the Local Research Ethics Committees, and the research carried out in accordance with the guidelines of both committees. Written informed consent was obtained from parents or a responsible legal guardian for the child to participate. Assent was obtained from the child participants where possible.

### Phenotypes

The DANVA is a series of computer administered assessments that measure the ability to decode nonverbal information, including facial expression and tone of voice. The ‘faces’ subtest comprises 24 photos of child faces, each showing one of four emotions: happy, sad, anger or fear. Each photo is presented on a computer screen for 2 seconds, during which time the child is prompted to respond whether they think the child portrayed is happy, sad, angry or fearful by clicking on the appropriate word presented on the screen below the image. Comprehension of meaning of the four words, ability to read them and understanding of the task itself is checked by an examiner before the child starts the assessment. From among those who completed the task (N = 7,303), 1,139 involved the tester recording the response on a datasheet (e.g. when there was technical difficulties). For the purpose of the current study, all unrelated children who completed the DANVA were included and then filtered to leave only those individuals with a maximum of one DANVA item missing. A sample of 4,919 children remained at this stage. As discussed subsequently, after further filtering based on the results of our population stratification analysis, a final sample of N = 4,780 children remained. All children also underwent IQ testing using the Wechsler Abbreviated Intelligence Scale (Pearson Clinical, London).

### Genotypes

GWAS data were generated by Sample Logistics and Genotyping Facilities at Wellcome Sanger Institute and LabCorp (Laboratory Corporation of America) using support from 23andMe. Genotyping was performed using the Illumina HumanHap550 quad chip genotyping platform. The resulting raw genome-wide data were subjected to standard quality control methods as described in the unpublished ALSPAC quality control document made available to researchers using these data. Individuals were excluded on the basis of gender mismatches; minimal or excessive heterozygosity; disproportionate levels of individual missingness (>3%) and insufficient sample replication (IBD < 0.8). Population stratification was assessed by multidimensional scaling analysis and compared with HapMap II (release 22) European descent (CEU), Han Chinese, Japanese and Yoruba reference populations; all individuals with non-European ancestry were removed. SNPs with a minor allele frequency of <1%, a call rate of <95% or evidence for violations of Hardy-Weinberg equilibrium (P < 5E-7) were removed. Cryptic relatedness was measured as proportion of identity by descent (IBD > 0.1). Related subjects that passed all other quality control thresholds were retained during subsequent phasing and imputation. 9,115 subjects and 500,527 SNPs passed these quality control filters.

### Imputation

ALSPAC mothers were genotyped using the Illumina human660W-quad array at Centre National de Génotypage (CNG) and genotypes were called with Illumina GenomeStudio. 477,482 SNP genotypes in common between the sample of mothers and sample of children were combined. SNPs with genotype missingness above 1% due to poor quality (11,396 SNPs removed) were removed, and a further 321 subjects were removed due to potential ID mismatches. This resulted in a dataset of 17,842 subjects containing 6,305 duos and 465,740 SNPs (112 were removed during liftover and 234 were out of HWE after combination). Haplotypes were estimated using ShapeIT (v2.r644)^43^ which utilises relatedness during phasing. Phased version of the 1000 genomes reference panel (Phase 1, Version 3) were obtained from the Impute2 reference data repository (phased using ShapeIt v2.r644^44^, haplotype release date Dec 2013). Imputation of the target data was performed using Impute V2.2.2^44^ against the reference panel (all polymorphic SNPs excluding singletons), using all 2,186 reference haplotypes (including non-Europeans). In total, 27,449,291 SNPs were tested (SNPS with MAF ≥ 0.01 = 9,128,173).

### Population structure

We performed an additional layer of population structure analysis. We first undertook analysis of population structure using the Roslin *et al*. (2016) QC’d 1000 Genomes phase 3 data as ref. ^45^. All non-imputed (i.e. Illumina) SNPs that are shared between ALSPAC and 1000 Genomes across the genome were used to investigate population structure after LD pruning (N = 106,084). After LD pruning, GCTA [v1.9.1]^46^ was used to generate a genetic relationship matrix which was in turn employed by GCTA to generate principal components. The first two eigenvectors thereby generated are visualized in the Supplementary Fig. 1 (Population structure for ALSPAC using Roslin *et al*. QC’d 1000 Genomes). Although the sample appears to cluster with the 1000 genomes EUR samples, we further investigated this by repeating the population stratification analysis against the EUR only 1000 genomes sample (Supplementary Fig. 2: Population structure for ALSPAC against 1000 Genomes EUR-only sample) which demonstrated some heterogeneity. Through a series of iterations, sequentially removing outliers and recalculating structure, a total of 222 ALSPAC individuals were removed and a well-clustered sample was generated for subsequent analyses (N = 4,780).

### LDSC

LD score regression (LDSC)^23^ was used also to investigate population stratification. This method, which regresses test statistic on variant LD scores across the genome under a polygenic model, provides estimation of heritability (slope) and confounding bias (intercept minus 1). With true polygenicity, the intercept approaches 1, but with population stratification the intercept shows positive deviation.

### Genome-wide association study

The final sample of 4,780 individuals underwent GWAS of autosomes using methods implemented in SNPTEST^47^ [v2.5.2]. The total DANVA score, indicating number of correct responses, was used as the quantitative trait of interest, with age and sex as covariates. DANVA scores were first cubed to normalize residuals [Supplementary Fig. 3a: distribution of DANVA scores in total sample; Supplementary Fig. 3b: distribution of DANVA-cubed scores in total sample]. Frequentist tests were performed for each SNP using an additive model, with a score test being employed to manage genotype uncertainty at each imputed SNP. Phenotypes were mean centred and scaled to have a variance of 1. QQ plots of p-values were generated, stratified according to allele frequency. The results are visually summarized in Manhattan plots and regional plots of top SNPs. In total, 27,449,291 SNPs were tested (SNPs with MAF ≥ 0.01 = 9,128,173).

### Generalized gene-set analysis

SNP P-values generated in the GWAS were then used in a genome-wide gene-by-gene analysis. Specifically, MAGMA [v.1]^25^ was used to undertake a gene-level linear regression. This method projects the matrix of overlapping SNPs for each gene (which will vary by gene) onto its principal components, which are then used as predictors for phenotype in a linear regression model. In the region of 15,000 genes are included in the model.

### Transcriptome-wide association analysis

A transcriptome-wide association (TWA) analysis was implemented in FUSION [V.1]^26^. Briefly, gene expression levels are imputed into the dataset based on the results of reference data in which gene expression and genotyping have both been performed. This imputation is based on the weights that have been assigned to SNPs in the reference data using a liner predictive model. Association can then be tested between trait and imputed expression. In this way, this method essentially examines how strongly variants associated with expression are also associated with the trait, and under certain circumstances has greater power than traditional SNP-based GWAS. We used GTEx whole blood (release V.6)^17^ for reference data using pre-computed weights by the FUSION development team.

#### Polygenic risk scores

Polygenic Risk Scores were calculated using methods implemented in PRSice-2^48^. Results from published GWAS studies of schizophrenia^27^, bipolar disorder^29^, educational attainment^30^, cognition^30^, ASD^27^ and obsessive-compulsive disorder^31^ were used for calculating scores. All scores were generated using the default average score statistic, and subsequent trait regression was performed with sex as covariant as with all other analyses. Clumping was performed in PRSice using the default r2 of 0.1 and 250 kb windows. Default PRSice p-value thresholds were used, and permutation using the default 10,000 iterations was used to generate an empirical p-value.

## Supplementary information

Supplementary Material.

Supplementary Dataset 1

Supplementary Dataset 2

Supplementary Dataset 3

## Data Availability

The datasets generated during and/or analysed during the current study are available for download on the journal website.
