# Supplementary material for: Genome-wide association study of emotional empathy in children
Source: Sci Rep. 2020 May 4;10:7469. doi: 10.1038/s41598-020-62693-6 (PMC7198552; doi:10.1038/s41598-020-62693-6)
Supplement: Supplementary file 1 — Supplementary Material. [file 41598_2020_62693_MOESM1_ESM.pdf]

Genome-wide association study of emotional empathy in children

Woodbury-Smith, MR[1,2 *], Paterson, AD[2,3], Szatmari, P[2,3,4], Scherer, SW[2,4,5]

Affiliations:

[1]Institute of Neuroscience, Newcastle University, Newcastle upon Tyne, UK.

[2]The Centre for Applied Genomics, The Hospital for Sick Children, Toronto, ON, Canada.

[3]Division of Epidemiology and Biostatistics, Dalla Lana School of Public Health, University of Toronto, Toronto, ON, Canada.

[4]Centre for Addiction and Mental Health, The Hospital for Sick Children & University of Toronto, Toronto, ON, Canada.

[5]McLaughlin Centre and Department of Molecular Genetics, University of Toronto, Toronto, ON, Canada.

Supplementary Material

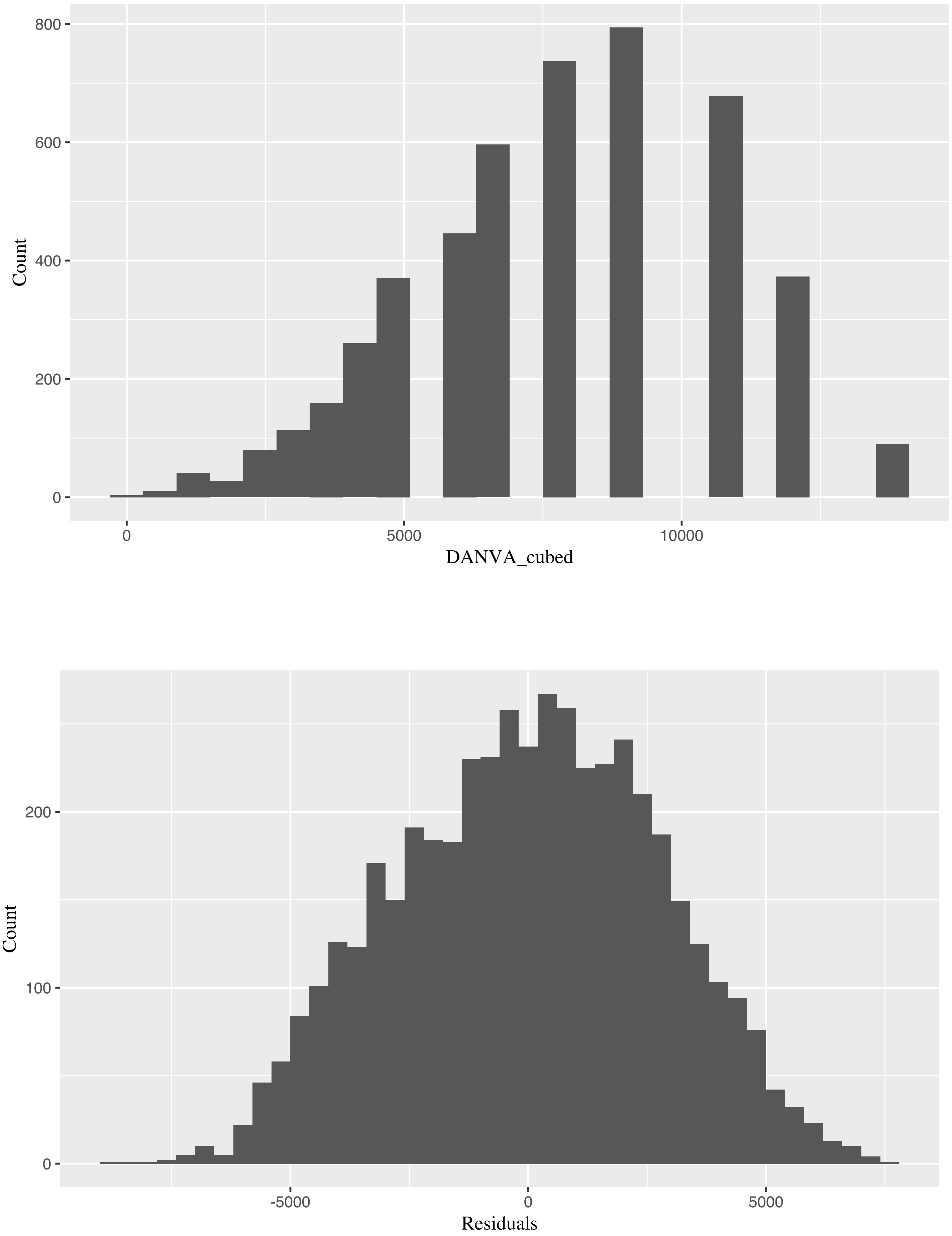

Supplementary Figure 1: Distribution of DANVA-cubed (top) and DANVA-cubed residualized for age, sex and IQ (bottom)

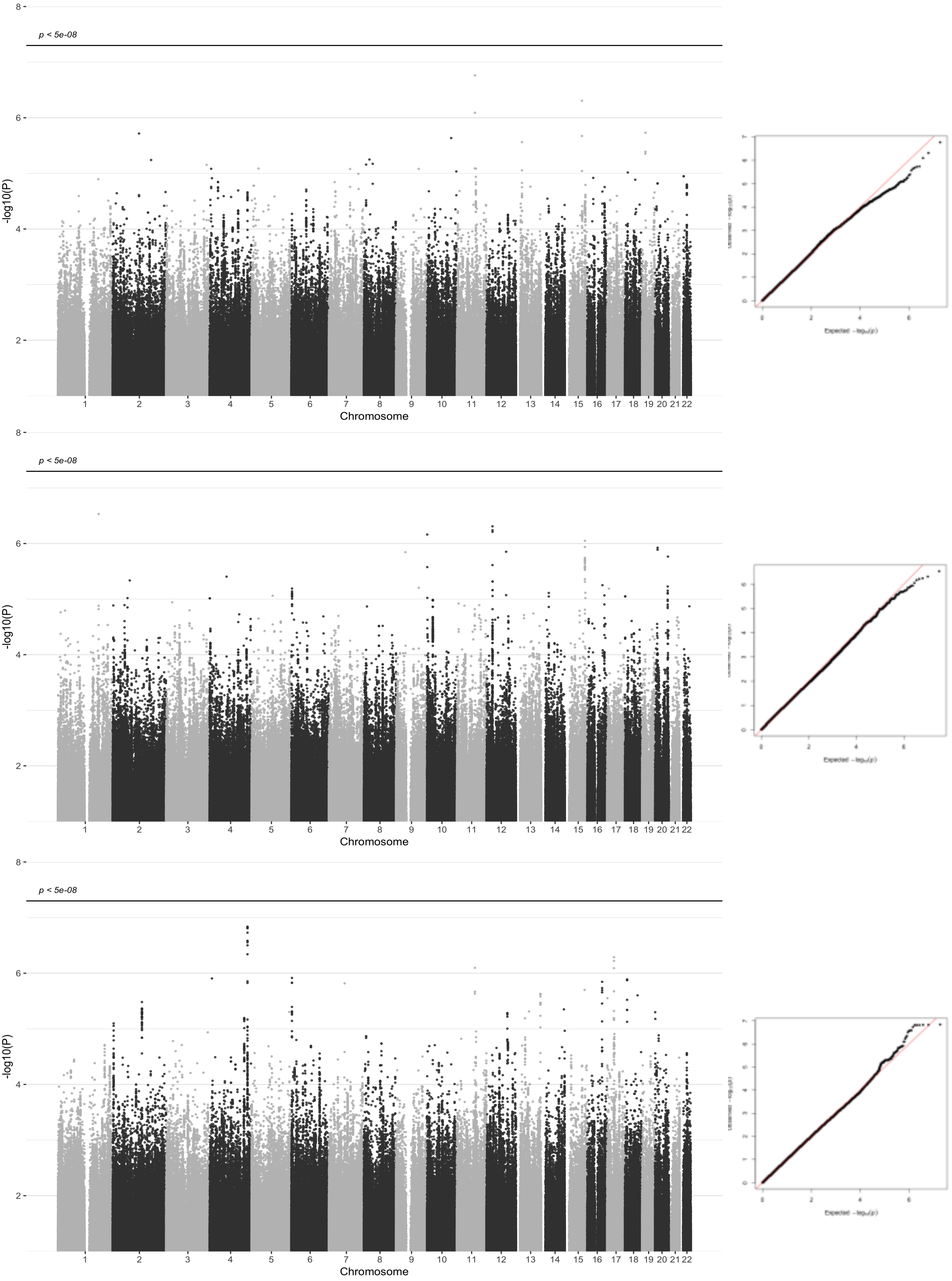

Supplementary Figure 2: Manhattan (left) and QQ (right) plots for GWAS (without IQ as covariate). Complete sample (top), female only (middle), male only (bottom). Note: y-axis starts at -log10(P)=1

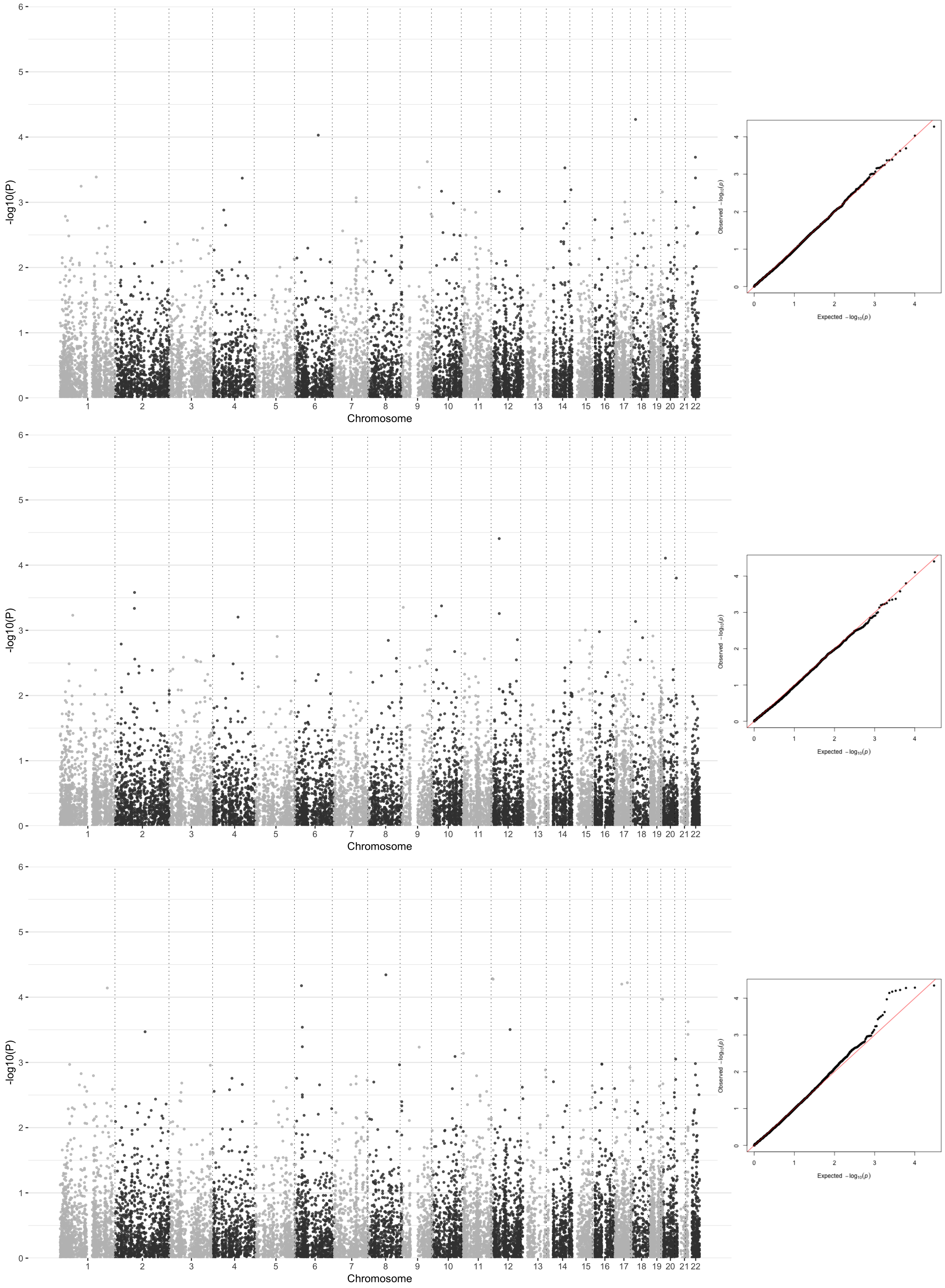

Supplementary Figure 3: Manhattan (left) and QQ (right) plots for gene based association. Complete sample (top), female only (middle), male only (bottom). Phenotype residualised for IQ, age and sex

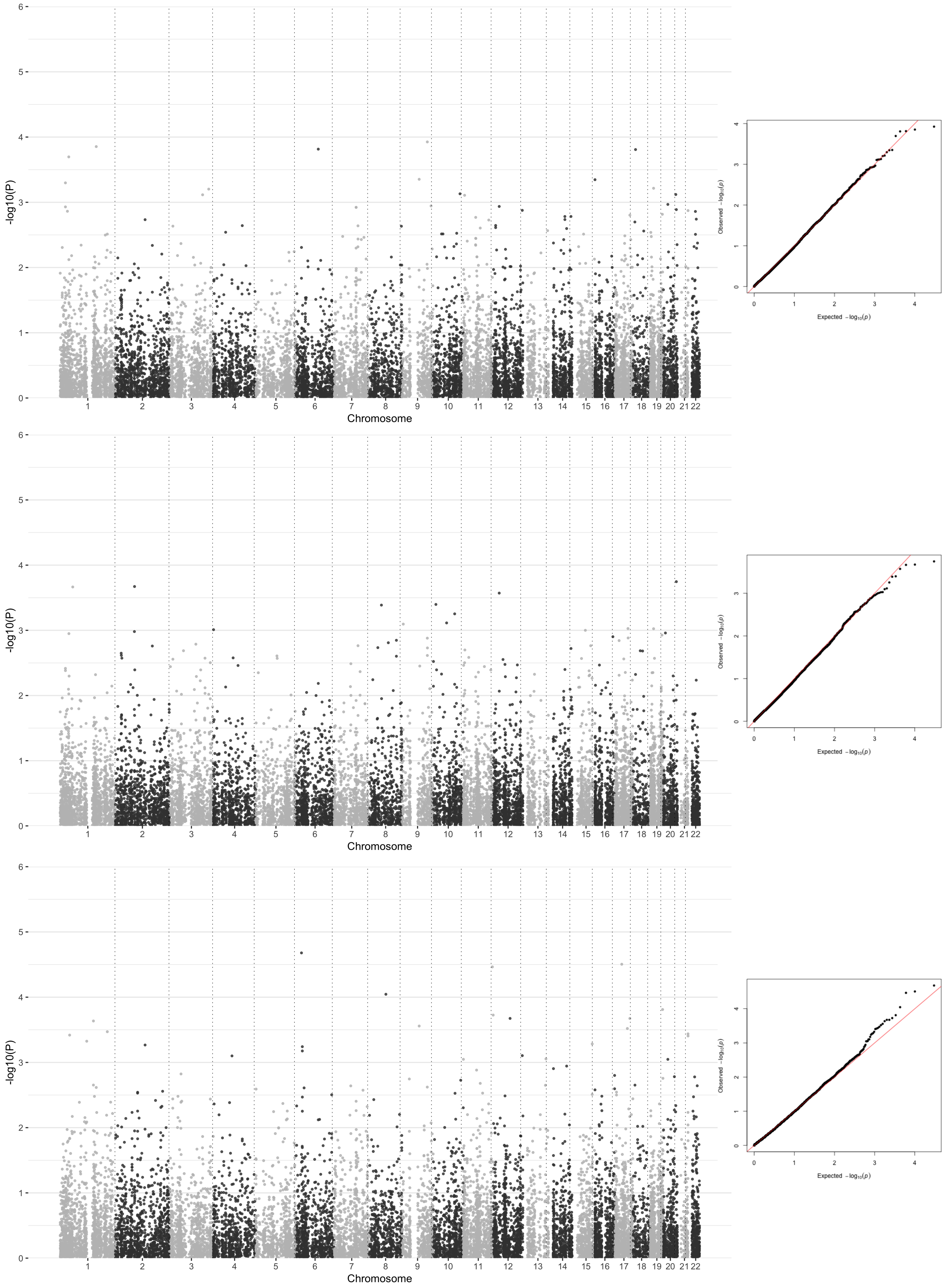

Supplementary Figure 4: Manhattan (left) and QQ (right) plots for gene based association. Complete sample (top), female only (middle), male only (bottom). Phenotype residualised for age and sex

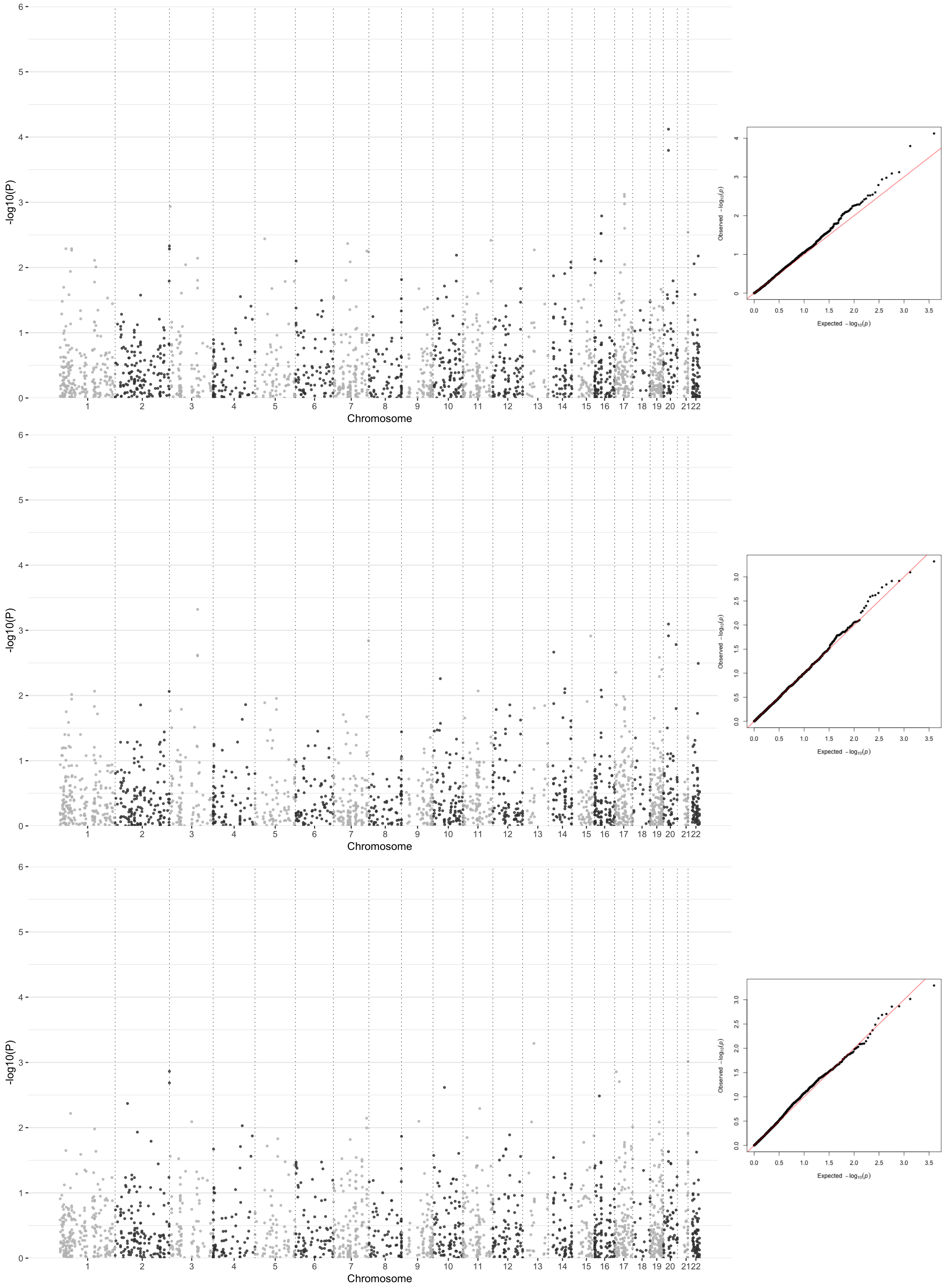

Supplementary Figure 5: Manhattan (left) and QQ (right) plots for TWAS, unstratified (top), female only (middle), male only (bottom). Phenotype residualised for IQ, age, sex

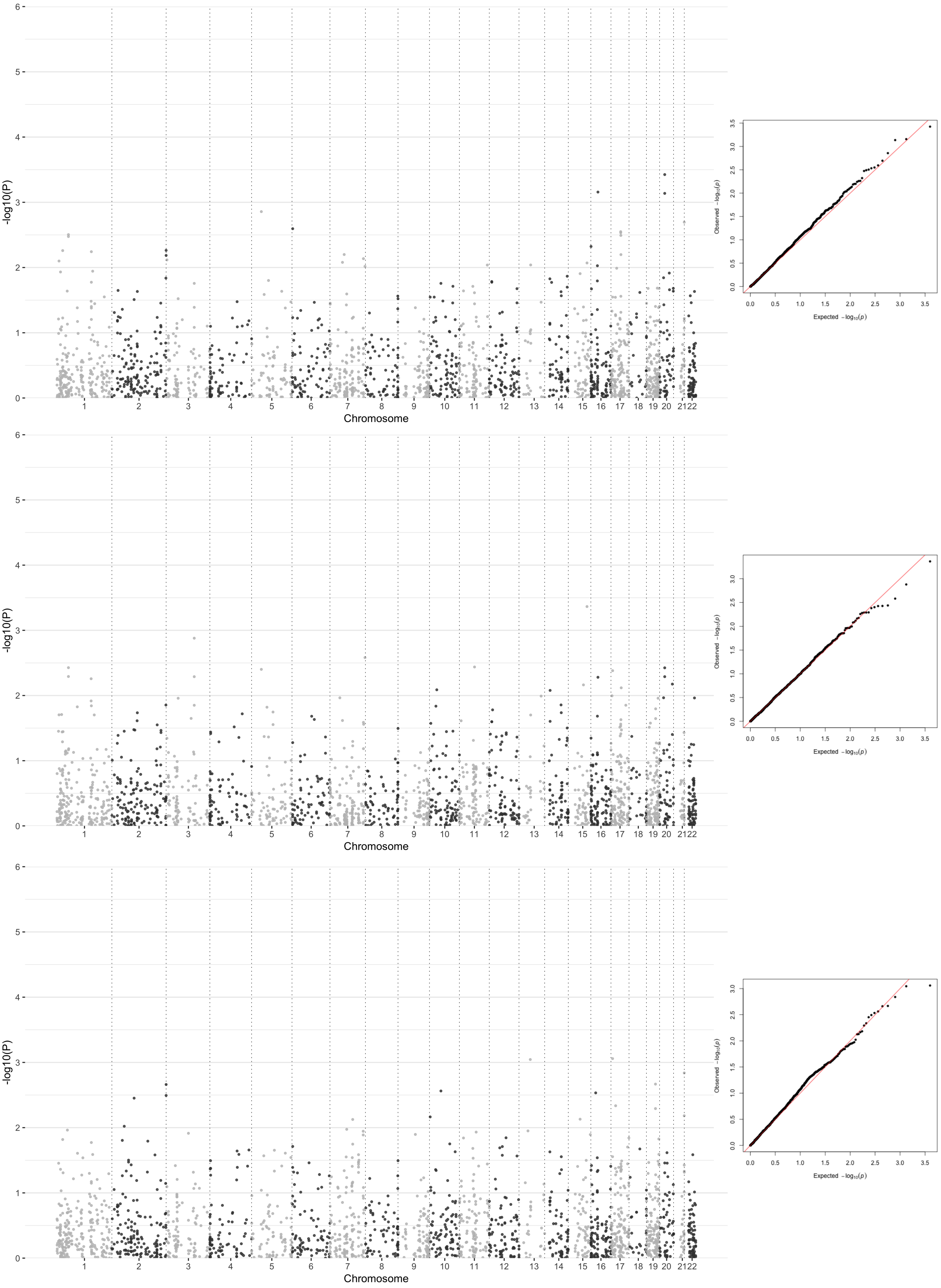

Supplementary Figure 6: Manhattan (left) and QQ (right) plots for TWAS, unstratified (top), female only (middle), male only (bottom). Phenotype residualised for age and sex.

Supplementary Table 1: Top SNPs from GWAS residualized for IQ, sex and age

CHR	SNP ID	BP	Allele 1	Allele 2	MAF*	Info	Beta	SE	P
Non-stratified									
5	rs12153376	10636348	C	T	0.0737043	1	543.2	108.99	6.44E-07
11	rs12790238	82199065	C	T	0.0162144	0.94	-1152.66	229.48	5.48E-07
19	rs28409744	14927401	G	A	0.45235	0.87	301.14	60.39	6.07E-07
Female only analysis									
1	rs12407722	185451121	G	A	0.0496475	0.73	-1151.37	212.72	5.61E-08
15	rs72761402	91597783	C	G	0.419443	0.99	394.44	80.04	8.86E-07
Male only analysis									
4	rs6553572	172401010	G	C	0.166867	0.99	-539.9	107.64	5.63E-07
4	rs12504961	172402306	A	G	0.151387	0.99	-565.51	112.33	5.14E-07
4	rs12505867	172402938	A	G	0.152732	1	-563.97	111.91	5.02E-07
4	rs7686071	172403576	T	C	0.152259	1	-564.53	111.88	4.84E-07
4	rs7683952	172407815	C	T	0.152063	1	-563.76	111.8	4.94E-07
4	rs12646964	172408167	C	T	0.168315	1	-536.88	107.29	6.01E-07
4	rs13435738	172408426	A	C	0.152061	1	-563.75	111.8	4.94E-07
4	rs1391207	172413977	A	G	0.152007	1	-563.39	111.81	5.03E-07
4	rs7693356	172414655	G	A	0.168606	0.99	-537.87	107.56	6.09E-07
4	rs17056945	172415689	A	T	0.168707	0.99	-537.11	107.23	5.82E-07
4	rs56045235	172416006	A	G	0.151989	0.99	-559.03	112.13	6.57E-07
7	rs113791338	71614217	G	A	0.0338594	0.94	-1134.17	224.19	4.79E-07
11	rs12790238	82199065	C	T	0.0161649	0.93	-1627.77	328.98	8.25E-07
13	rs67357970	111549458	T	G	0.123144	0.97	615.76	125.4	9.69E-07
13	rs13343	111549790	C	T	0.123145	0.96	615.74	125.41	9.71E-07
13	rs66681527	111550791	C	T	0.123152	0.96	615.66	125.41	9.75E-07
13	rs147595846	111555680	GTTTTAA	G	0.123173	0.96	615.57	125.45	9.86E-07
14	rs144308953	103229433	C	A	0.0158311	0.65	-1819.48	367.59	8.79E-07
16	rs117467133	67572534	G	A	0.0196945	0.9	1526.15	311.24	9.86E-07
16	rs118187571	67616809	C	T	0.0193643	0.89	1564.28	315.97	7.58E-07
17	rs2729340	30996326	G	A	0.473182	0.99	404.18	80.21	5.02E-07
17	rs2032753	31000816	G	T	0.472439	0.99	410.99	80.2	3.21E-07
17	rs2640840	31004275	G	A	0.475519	0.99	413.48	80.34	2.87E-07

Note: * allele 2 is minor allele; CHR=chromosome; BP is basepair position (GRCh37); Info=imputation (value 1 indicates SNP with high certainty).

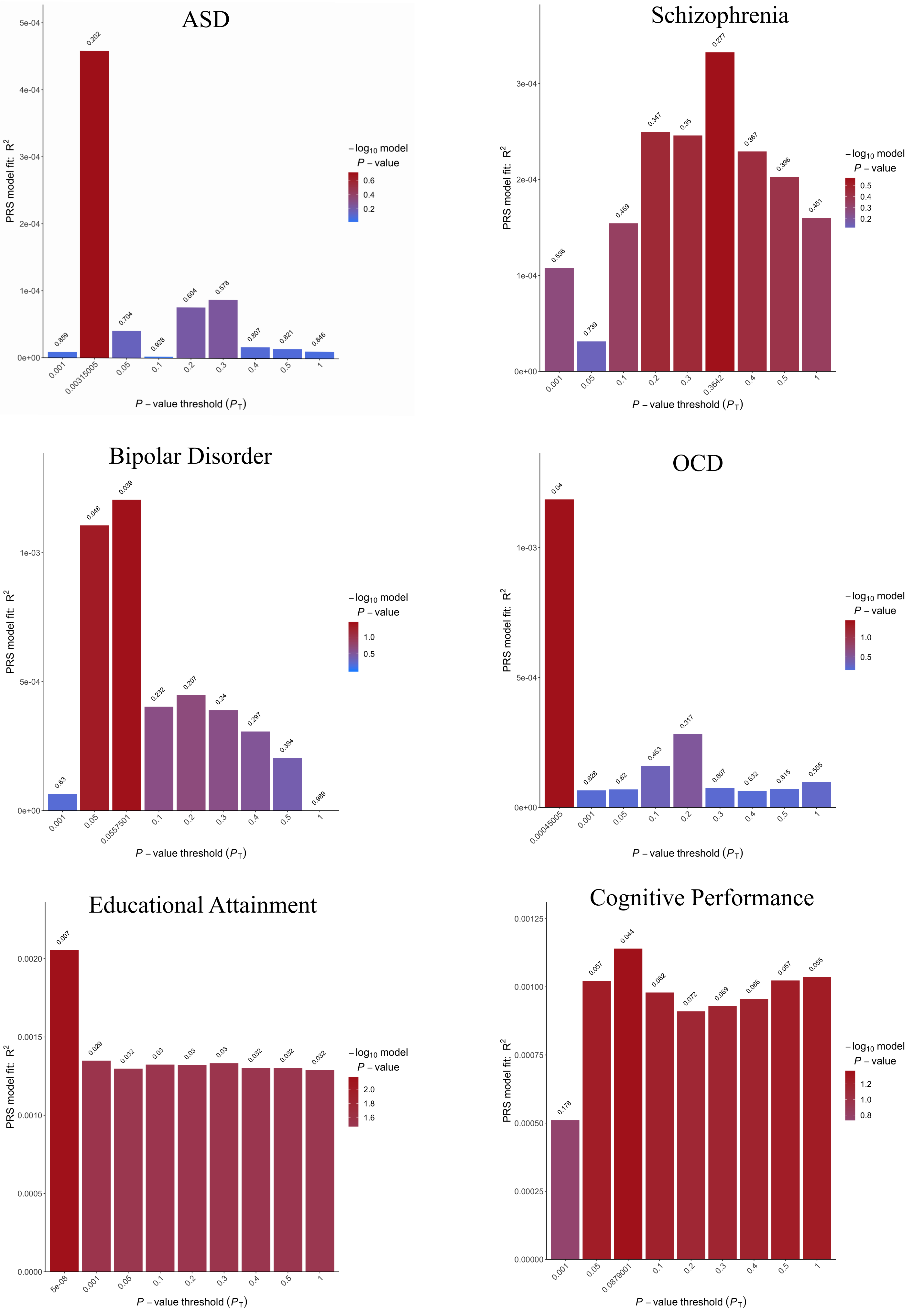

Supplementary Figure 7: Poygenic risk score (y-axis, model fit) with different base datasets across different p-value thresholds (x-axis).

Supplementary Table 2: Top SNPs from GWAS residualized for sex and age

CHR	SNP ID	BP	Allele 1	Allele 2	MAF*	Info	Beta	SE	P
Non-stratified									
3	rs181330121	105669986	A	C	0.00320835	0.72	-2961.09	582.52	2.75E-07
11	rs12790238	82199065	C	T	0.0161055	0.94	-1233.13	235.27	1.72E-07
11	rs76447514	82232867	A	G	0.016841	0.94	-1141.49	231.18	8.13E-07
15	rs144294187	77948530	A	T	0.0068638	0.68	-2097.32	415.25	5.44E-07
15	rs140742336	77973408	C	T	0.00971206	0.82	-1620.07	321.12	4.94E-07
Female only analysis									
1	rs12407722	185451121	G	A	0.0494114	0.73	-1113.22	218.16	2.94E-07
10	rs200108031	814601	AC	A	0.0119025	0.69	-2215.73	455.49	6.88E-07
12	rs1907652	27172130	A	T	0.309608	0.96	1113.58	792	5.76E-07
12	rs11048818	27172652	A	G	0.30721	0.99	-443.21	87.87	4.89E-07
12	rs12825917	27411600	G	A	0.125992	1	613.52	122.72	6.16E-07
15	rs72761402	91597783	C	G	0.418802	0.99	403.4	81.89	8.93E-07
Male only analysis									
4	rs6553572	172401010	G	C	0.166373	0.99	-561.29	109.47	3.15E-07
4	rs12504961	172402306	A	G	0.151144	0.99	-600.88	114.19	1.54E-07
4	rs12505867	172402938	A	G	0.152476	0.99	-598.69	113.77	1.55E-07
4	rs7686071	172403576	T	C	0.15201	1	-599.68	113.72	1.46E-07
4	rs10014387	172406840	C	T	0.153068	1	-574.06	113.52	4.56E-07
4	rs7683952	172407815	C	T	0.151819	1	-598.71	113.66	1.50E-07
4	rs12646964	172408167	C	T	0.167797	1	-562.17	109.12	2.78E-07
4	rs13435738	172408426	A	C	0.151816	1	-598.69	113.66	1.50E-07
4	rs1391207	172413977	A	G	0.151763	1	-598.17	113.66	1.54E-07
4	rs7693356	172414655	G	A	0.168118	0.99	-564.34	109.32	2.62E-07
4	rs17056945	172415689	A	T	0.168186	0.99	-562.95	109.05	2.62E-07
4	rs56045235	172416006	A	G	0.15174	0.99	-595.68	113.99	1.87E-07
11	rs12790238	82199065	C	T	0.0159151	0.93	-1671.96	337.58	7.99E-07
17	rs2729340	30996326	G	A	0.47343	0.99	403.22	81.53	8.10E-07
17	rs2032753	31000816	G	T	0.472712	0.99	407.93	81.52	6.01E-07
17	rs2640840	31004275	G	A	0.475761	0.98	411.16	81.68	5.15E-07

Note: * allele 2 is minor allele; CHR=chromosome; BP is basepair position (GRCh37); Info=imputation (value 1 indicates SNP with high certainty).

Supplementary Table 3: Comparison of current study with Coleman et al. (2017)

	Current study	Coleman et al.
Sample size	4,780	4,097
Exclusion	<23 DANVA items completed	<23 DANVA items completed; diagnosed ASD; IQ<70
DANVA transformation	Cubed	Arcsine
Covariates	Sex, age; Sex, age, IQ	Sex, age, IQ, test order
Imputation	1000 Genomes Phase 1, Version 3	HapMap2, release 22
Population stratification	Iterative PCA	None
Post hoc analyses	Polygenic Risk Score; Gene-based association; Transcriptome-wide association	Polygenic Risk Score
